# A Metastasizing Squamous Cell Carcinoma Arising in a Solitary Epidermal Nevus

**DOI:** 10.1155/2012/109632

**Published:** 2012-08-01

**Authors:** Masami Toya, Yuichiro Endo, Akihiro Fujisawa, Miki Tanioka, Yoshiaki Yoshikawa, Takao Tachibana, Yoshiki Miyachi

**Affiliations:** ^1^Department of Dermatology, Graduate School of Medicine, Kyoto University, 54 Shogoin-Kawara-cho, Sakyo, Kyoto 606-8507, Japan; ^2^Department of Dermatology, Otsu Red Cross Hospital, 1-1-35 Nagara, Otsu-shi, Shiga 520-8511, Japan; ^3^Department of Dermatology, Osaka Red Cross Hospital, 5-30 Fudegasaki-cho, Tennoji-ku, Osaka 543-8555, Japan

## Abstract

*Aim*. Secondary tumor rarely develops from epidermal nevus. We present a case of a metastasizing squamous cell carcinoma that developed in a solitary epidermal nevus. *Case Report*. An 82-years old Japanese female was presented with a red tumor on the left axilla. She reported that the tumor developed in a congenital epidermal nevus. A biopsy of the tumor showed that a well-differentiated squamous cell carcinoma (SCC) arose from the epidermal nevus. As a lymph node metastasis was found by sentinel lymph node biopsy, the patient received surgical excision of the lesion, axillary lymph node dissection, and postoperative radiation. *Discussion*. Secondary tumors developing in epidermal nevus are rare. To the best of our knowledge, only in two cases including the present case, SCC developed in a solitary epidermal nevus. There is no established clinical guideline for prophylactic removal of epidermal nevus. However, a biopsy should be done if a secondary malignancy is suspected in an epidermal nevus.

## 1. Introduction

The presence of secondary tumors in various types of congenital nevi is well known; however, malignancy associated with epidermal nevi is rare. Here, we present a case of a metastasizing squamous cell carcinoma that developed in a solitary epidermal nevus. 

## 2. Case Report

**Figure 1:**
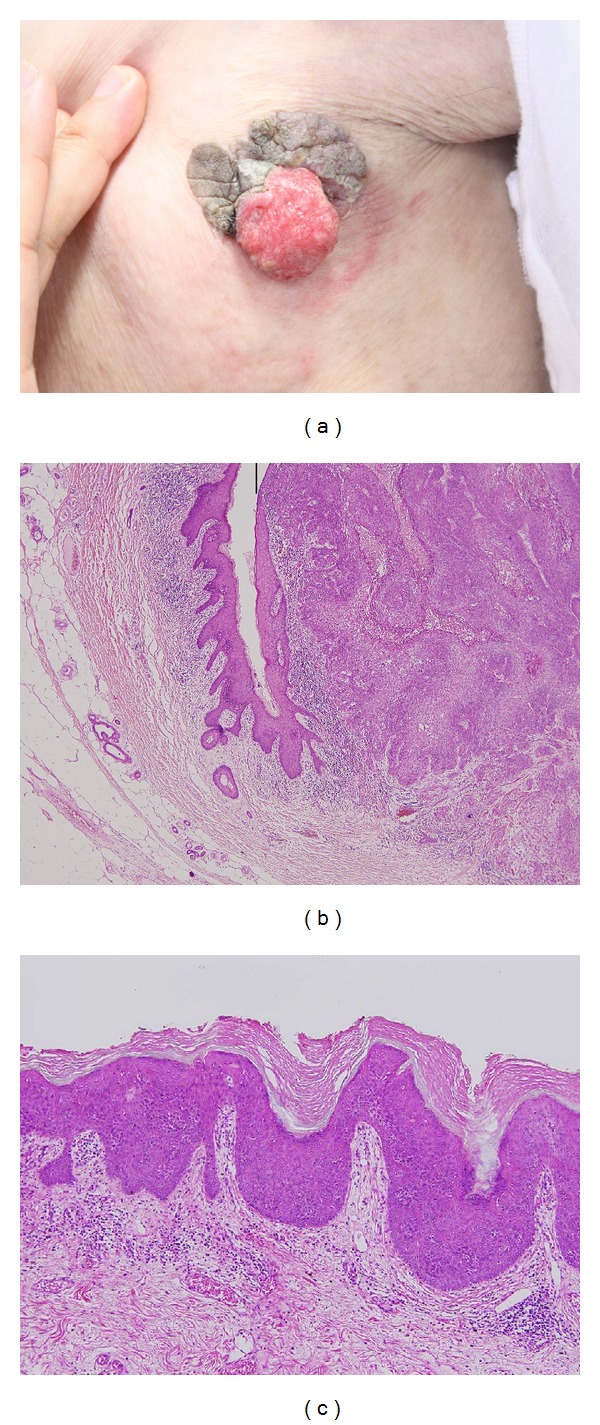
(a) A red tumor on the left axilla developed in a congenital verrucous plaque. (b) SCC lesion (left side of the vertical bar) developed within the epidermal nevus (hematoxyline-eosin stain, original magnification: ×40). (c) Verrucous proliferation of the keratocyte and partial granular degeneration which were compatible with a finding of an epidermal nevus (hematoxyline-eosin stain, ×100).

 An 82-year-old Japanese female presented with a red tumor on the left axilla. She reported that the tumor developed in a verrucous plaque that had existed since birth. The size of the tumor was 25 mm in diameter, and the plaque was 45 × 40 mm (Figure 1(a)). A biopsy of the tumor showed that it was a well-differentiated squamous cell carcinoma (SCC). Surgical excision of the tumor and a sentinel lymph node biopsy of the right axilla were performed. Pathological examination showed that the SCC had invaded the subcutaneous fat tissue. The SCC developed within a papillomatous epidermal lesion showing hyperkeratosis and acanthosis (Figures 1(b) and 1(c)). The sentinel lymph node was positive for tumor cell; therefore, the patient underwent a subsequent axillary lymph node dissection and 60 Gray of postoperative radiation. Seven out of 30 excisional lymph nodes were positive for tumor cells. A computed tomography scan detected no metastasis to internal organs. We diagnosed the tumor as pT2N2bM0, stage IV (UICC 7th edn.). The patient was tumor-free for a year after the axillary lymph node dissection.

## 3. Discussion

 Secondary tumors developing in epidermal nevi are rare. Only a few reports had described secondary cutaneous tumors deriving from epidermal nevi such as basal cell carcinoma, SCC, Bowen's disease, malignant eccrine poroma, keratoacanthoma, clear cell acanthoma, and trichoepithelioma [1]. To the best of our knowledge, only seven cases of secondary SCC have been reported in the last 30 years [2–4]. In five cases, tumors arose on multiple or linear epidermal nevi. Only in two cases, including the current case, a SCC developed in a solitary epidermal nevus [5].

In cutaneous SCC, tumor size, gender, preceding lesions, histological findings such as the degree of the differentiation, and location of tumors have been reported as prognostic factors of local recurrence, metastasis, and disease-specific death [6–10]. In this case, the tumor location was on the trunk, the differentiation was good, and the tumor size was smaller than 2 cm, which meant that this case had a lower risk of metastasis. It may be that the epidermal nevi made it harder for the patient to notice the SCC lesion and gave enough time for the SCC to metastasize. 

 There is no established clinical consensus for the prophylactic removal of epidermal nevi. However, the rate of secondary malignancy in sebaceous nevi, which are classified into epithelial nevi such as epidermal nevus, was reported to be 14%, with a malignant change occurring in 0.8% [11]. Therefore, one paper suggests that the prophylactic removal of sebaceous nevi is not necessary because sebaceous nevi are at low risk of developing secondary malignant tumors, and its prognosis tends to be good even when a malignant tumor develops from the lesion [11]. A similar strategy can be applied to the prophylactic removal of epidermal nevi. A biopsy should be considered if a change in an epidermal nevi is noticed.
